# Associations of ECG-derived markers with clinical and biological characteristics in systemic sclerosis: preliminary findings from the SCAR-SSc study

**DOI:** 10.25122/jml-2026-0092

**Published:** 2026-09

**Authors:** Cristina Andreea Vrancianu, Silvia Gabriela Arbanasu, Dragos Emanuel Popa, Bianca Budu, Isa Ethem Demirkol, Oana Andreea Popa, Adriana Iliesiu, Mihai Bojinca, Ana Ciobanu, Ana Maria Gheorghiu

**Affiliations:** 1 Faculty of Medicine, Carol Davila University of Medicine and Pharmacy, Bucharest, Romania; 2 Department of Internal Medicine and Rheumatology, Cantacuzino Clinical Hospital, Bucharest, Romania; 3 Department of Cardiology, Theodor Burghele Clinical Hospital, Bucharest, Romania; 4 Faculty of Medicine, Saarland University, Saarbrücken, Germany; 5 Department of Cardiology, Prof. Dr. Agrippa Ionescu Emergency Clinical Hospital, Ilfov, Romania

**Keywords:** scleroderma, systemic, electrocardiography, heart conduction system, ventricular premature complexes, natriuretic peptide, brain, electrocardiography, ambulatory, biomarkers, ACA, anti-centromere antibodies, ACR, American College of Rheumatology, AF, atrial fibrillation, ANA, antinuclear antibodies, ATA, anti-topoisomerase I antibodies, BNP, B-type natriuretic peptide, CI, confidence interval, CRP, C-reactive protein, CT, computed tomography, dcSSc, diffuse cutaneous systemic sclerosis, DLCO, diffusing capacity of the lung for carbon monoxide, DU, digital ulcers, ECG, electrocardiogram, EQ-5D-5L, EuroQol 5-dimension 5-level questionnaire, EULAR, European Alliance of Associations for Rheumatology, fQRS, fragmented QRS, HRCT, high-resolution computed tomography, hsTnI, high-sensitivity troponin I, IAB, interatrial block, IQR, interquartile range, lcSSc, limited cutaneous systemic sclerosis, mRSS, modified Rodnan skin score, MVP, morphology-voltage-P-wave duration, NT-proBNP, N-terminal pro-B-type natriuretic peptide, OR, odds ratio, PFT, pulmonary function test, PROM, patient-reported outcome measure, PVC, premature ventricular contraction, PWD, P-wave duration, PWVI, P-wave voltage in lead I, Sclero-ID, EULAR Systemic Sclerosis Impact of Disease, SHAQ, Scleroderma Health Assessment Questionnaire, SSc, systemic sclerosis, ST2, suppression of tumorigenicity 2

## Abstract

Cardiac involvement in systemic sclerosis (SSc) is often subclinical in its early stages. Electrocardiographic markers have been associated with arrhythmias and myocardial remodeling and may provide insight into cardiac involvement in SSc. This pilot single-center cross-sectional study explored associations between ECG-derived parameters and clinical and biological features in patients with SSc without overt cardiovascular disease. Thirty-four patients meeting the 2013 American College of Rheumatology/European Alliance of Associations for Rheumatology classification criteria were included. N-terminal pro-B-type natriuretic peptide (NT-proBNP) and high-sensitivity troponin I were measured, standard 12-lead ECGs were analyzed for interatrial block (IAB), morphology-voltage-P-wave duration (MVP) score and fragmented QRS (fQRS), and 24-hour Holter monitoring was used to quantify arrhythmias. Participants were 85.3% women, with a median (IQR) age of 53.6 (46–61) years and disease duration of 7.95 (1.5–14.8) years; 76.5% had limited cutaneous SSc. Fragmented QRS was present in 7 of 32 patients with evaluable fQRS data (21.9%), and its presence was associated with a higher premature ventricular contraction count on Holter monitoring after adjustment for age and sex (OR = 1.0022; 95% CI, 1.0001–1.0044; *P* = 0.044). Partial IAB was present in 4 of 8 patients (50%) with diffuse cutaneous SSc. The modified Rodnan skin score (mRSS) differed between patients with and without IAB (9 ± 9 versus 3 ± 4, *P* = 0.040). Higher MVP scores were associated with mRSS (β = 0.422, *P* = 0.026), C-reactive protein (β = 0.412, *P* = 0.028) and NT-proBNP (β = 0.557, *P* = 0.003). ECG-derived markers were associated with measures of disease severity, ventricular ectopy and NT-proBNP levels. These exploratory findings require confirmation in larger studies.

## INTRODUCTION

Systemic sclerosis (SSc) is a chronic autoimmune disease in which microvascular damage and fibrosis of the skin and internal organs are the main features. When the heart is involved, these changes can lead to arrhythmias, ventricular dysfunction, and heart failure. Cardiac involvement is often subclinical in the early stages and may remain undetected and untreated, leading over time to significant morbidity and mortality. When cardiac symptoms develop, they are often nonspecific and may include dyspnea, palpitations, and chest pain resembling angina; these manifestations can result from both primary cardiac involvement and secondary complications. Atrial and ventricular arrhythmias, as well as conduction disturbances of varying severity, are common complications in SSc [1]. Studies have shown that conduction abnormalities occur in approximately 20% to 33% of patients with SSc, with left bundle branch block being the most common, followed by first-degree atrioventricular block [1–3]. Cardiac biomarkers such as B-type natriuretic peptide (BNP) and N-terminal pro-B-type natriuretic peptide (NT-proBNP), which are released in response to cardiac wall stress, are established markers of heart failure, and elevated levels are associated with poor prognosis in SSc. These peptides can rise in both right and left ventricular dysfunction, and NT-proBNP is considered an independent predictor of mortality in SSc [4]. Other biomarkers, such as troponin and soluble suppression of tumorigenicity 2 (ST2), may reflect myocardial injury or fibrosis and offer enhanced prognostic value when used in combination [5].

Myocardial inflammation and fibrosis may affect both atrial and ventricular conduction pathways before overt cardiac manifestations become clinically evident. Several ECG-derived markers have been associated with arrhythmias [6–8]. The morphology-voltage-P-wave duration (MVP) score combines P-wave duration (PWD), P-wave voltage in lead I (PWVI), and interatrial block (IAB), while fragmented QRS (fQRS) indicates inhomogeneous ventricular conduction. Fragmented QRS complexes have been reported more frequently in patients with SSc than in the general population, and their presence has been linked to heterogeneous ventricular conduction, myocardial scar formation, and an increased risk of arrhythmias [9].

Although data in SSc are scarce, IAB and MVP are generally associated with atrial fibrosis and electrical remodeling, with advanced IAB carrying a higher incidence of arrhythmias than partial forms [10,11]. Given that myocardial fibrosis represents a central mechanism of cardiac involvement in SSc, these ECG-derived markers may be associated with electrical changes related to myocardial remodeling.

This pilot study aimed to explore associations between ECG-derived parameters and clinical and biological features in patients with SSc without overt cardiovascular disease.

## MATERIAL AND METHODS

Consecutive patients fulfilling the American College of Rheumatology (ACR)/European Alliance of Associations for Rheumatology (EULAR) 2013 classification criteria for systemic sclerosis were recruited from the Department of Internal Medicine and Rheumatology, Cantacuzino Clinical Hospital, between October 2022 and November 2024. The present analysis was conducted within the SCAR-SSc (Subclinical CARdiac involvement in Systemic Sclerosis) study. The study was not prospectively registered in a clinical study registry. All patients underwent extensive rheumatological and cardiological evaluation.

### Exclusion criteria

Patients were excluded if they had known chronic coronary artery disease, cardiac structural abnormalities, significant valvular disease, a history of deep vein thrombosis or pulmonary embolism, pulmonary hypertension, acute or chronic respiratory failure, chronic kidney disease stages III–V, malignancy, atrial fibrillation, bundle branch block, preexcitation syndrome, electrolyte imbalance, or antiarrhythmic drug therapy at the time of inclusion.

### Rheumatological evaluation

During the rheumatological evaluation, information on disease history and current symptoms was collected and a clinical examination was performed. In addition, each participant completed the SSc Health Assessment Questionnaire (SHAQ) [12], the EULAR Systemic Sclerosis Impact of Disease (Sclero-ID) questionnaire [13] and the EuroQol 5-dimension 5-level (EQ-5D-5L) questionnaire [14]. Routine laboratory tests were performed: blood count, biochemistry (glycemia, creatinine, transaminases) and specific immunological tests including antinuclear antibodies (ANA) and an extended ANA profile. Patients underwent pulmonological evaluation with chest radiography and/or high-resolution computed tomography (HRCT), as well as pulmonary function tests and diffusing capacity of the lung for carbon monoxide, annually or earlier if needed, as part of routine assessment according to current recommendations [15].

### Cardiological evaluation

Patients were evaluated with a resting 12-lead electrocardiogram (BTL-08 Elite, BTL Industries Limited, Stevenage, United Kingdom) in the supine position. Tracings were recorded at a sampling rate of 1000 Hz with a low-pass filter of 150 Hz, and measurements were made using dedicated software (BTL CardioPoint). Recordings acquired with filter settings that differed from those specified in the study protocol were excluded from the fragmented QRS (fQRS) analysis. For measurement accuracy, ECG tracings were digitally magnified within the software by increasing the display paper speed to 150 mm/s and the voltage to 40 mm/mV. All ECG recordings were analyzed by the same cardiologist, who was blinded to the patients’ clinical data and laboratory results. Each ECG was evaluated once. In borderline cases, the ECG was reviewed by a second cardiologist, also blinded to the clinical and laboratory data. The anatomical distribution of fQRS (inferior, anterior, lateral, inferolateral and anterolateral) was recorded. Inter- and intra-observer agreement was not formally assessed.

The following parameters were determined: heart rate, P-wave duration, PR interval, QRS duration, QT interval, the presence of interatrial block, the MVP score and QRS fragmentation.

MVP score, fQRS and IAB were selected a priori as ECG markers of interest, based on previously reported associations with atrial and ventricular conduction abnormalities and arrhythmic risk. Their evaluation in SSc was exploratory, and the present study was not designed to validate these ECG markers as diagnostic markers of cardiac involvement.

Interatrial block was classified as advanced when a biphasic P wave in the inferior leads had a duration ≥120 ms, partial when a non-biphasic P wave in the inferior leads had a duration ≥120 ms, and absent when a non-biphasic P wave in the inferior leads had a duration <120 ms [16]. The MVP score was calculated from measurements obtained on the digitally magnified tracings in BTL CardioPoint [11]. Each of the three components was assigned 0 to 2 points: P-wave morphology in the inferior leads (non-biphasic <120 ms = 0, non-biphasic ≥120 ms = 1, biphasic = 2), P-wave voltage in lead I (>0.20 mV = 0, 0.10–0.20 mV = 1, <0.10 mV = 2) and P-wave duration (<120 ms = 0, 120–140 ms = 1, >140 ms = 2). The three components were summed to give a total MVP score ranging from 0 to 6.

Fragmented QRS complexes were identified on the standard 12-lead ECG by the presence of an additional R wave (R′) or by notching of the R or S wave within QRS complexes of duration <120 ms, observed in at least two adjacent leads. In patients with wide QRS complexes (≥120 ms), fragmentation was defined by the occurrence of multiple (>2) notches within the R or S wave [17]. ECGs with fewer than 10 interpretable leads or fewer than 5 interpretable precordial leads, and ECGs with U waves, were excluded from the analysis.

All patients underwent 24-hour Holter ECG monitoring using the Labtech EC-12H Holter ECG system (Labtech Ltd., Debrecen, Hungary) for the assessment of arrhythmic events. The absolute number of premature ventricular contractions (PVCs) during the recording was quantified. Automated analysis was followed by manual review by a cardiologist, with manual exclusion of artifacts, motion-related noise and atrial ectopic beats from PVC quantification. Complex ventricular ectopy was defined by the presence of ventricular bigeminy, trigeminy, couplets or nonsustained ventricular tachycardia during the 24-hour recording. Atrial tachycardia episodes identified on Holter analysis were reviewed and confirmed by a cardiologist. Echocardiography was performed in all patients as part of the cardiovascular evaluation and was used in the present analysis to identify patients with pulmonary hypertension and structural cardiac disease, who were excluded according to the predefined eligibility criteria.

### Statistical analysis

Statistical analysis was performed using IBM SPSS Statistics version 22 (IBM Corp., Armonk, NY, USA). As this was an exploratory pilot study, no formal sample size calculation was performed.

Group comparisons were performed using the independent samples *t* test or the Mann–Whitney U test, as appropriate. Categorical variables were compared using the chi-square test or Fisher’s exact test. Binary logistic regression was used to estimate associations, expressed as odds ratios (ORs) with 95% confidence intervals (CIs), with multivariable models adjusted for age and sex. Correlations between continuous variables were determined using Spearman’s rank correlation coefficient. Linear regression was used to assess associations between variables, with additional models adjusted for age and sex to control for potential confounding. A nominal *P* value <0.05 was considered statistically significant.

For each regression analysis, the variable of interest was entered together with age and sex in the same model, and separate models were fitted for each variable of interest. Interaction terms were not tested because of the limited sample size. Age and sex were selected a priori as adjustment variables, as clinically relevant demographic covariates, while limiting the number of covariates to avoid overfitting in this small exploratory cohort. Consequently, residual confounding by variables not included in the models cannot be excluded. Given the exploratory nature of the study, no adjustment for multiple comparisons was applied; all reported *P* values are nominal and should be interpreted as exploratory rather than confirmatory.

Missing values were coded as missing, and analyses were performed using complete-case methodology (listwise deletion).

## RESULTS

A total of 90 patients with systemic sclerosis were screened between October 2022 and November 2024. Of these, 35 met the eligibility criteria and were evaluated; the majority of those screened were ineligible, mainly because of the predefined exclusion conditions (structural cardiac disease, pulmonary hypertension, significant comorbidities). One patient was subsequently excluded following a newly diagnosed malignancy, leaving 34 patients for the final analysis. Baseline demographic, clinical, and serological characteristics and cardiovascular risk factors of the cohort are summarized in Table 1. The cohort had a median age of 53.6 years and a median disease duration of 7.95 years. Digital ulcers were present in 44.1% of patients. High-sensitivity troponin I (hsTnI) levels were within the normal range in all patients.

**Table 1:** Baseline characteristics of the study population (*n* = 34)

Characteristic	Value
**Demographic characteristics**	
Age, years, median (IQR)	53.6 (46–61)
Female, *n* (%)	29 (85.3)
Disease duration, years, median (IQR)	7.95 (1.5–14.8)
**Clinical features**	
History of digital ulcers, *n* (%)	15 (44.1)
Active digital ulcers, *n* (%)	10 (29.4)
dcSSc, *n* (%)	8 (23.5)
mRSS, median (IQR)	4 (0–6)
Telangiectasias, *n* (%)	25 (73.5)
**Biomarkers**	
NT-proBNP, pg/mL, median (IQR)	123 (74–234)
hsTnI, ng/L, median (IQR)	3 (2–4)
CRP, mg/L, median (IQR)	3 (1–6)
**Autoantibody profile**	
ATA, *n* (%)	15 (44.1)
ACA, *n* (%)	13 (38.2)
**Cardiovascular risk factors**	
Dyslipidemia, *n* (%)	18 (52.9)
Arterial hypertension, *n* (%)	10 (29.4)
Diabetes mellitus, *n* (%)	3 (8.8)
Overweight, *n* (%)	12 (35.3)
Obesity, *n* (%)	3 (8.8)
Current smokers, *n* (%)	5 (14.7)
Former smokers, *n* (%)	7 (20.6)
**Immunosuppressive therapy**	
Methotrexate, *n* (%)	9 (26.5)
Azathioprine, *n* (%)	8 (23.5)
Mycophenolate mofetil, *n* (%)	2 (5.9)
Tocilizumab, *n* (%)	1 (2.9)
No immunosuppressive therapy, *n* (%)	14 (41.2)
**Holter findings**	
Atrial tachycardia, *n* (%)	6 (17.6)
Complex ventricular ectopy, *n* (%)	6 (17.6)
Supraventricular ectopic beats, median (IQR)	26.5 (5–144)
Ventricular ectopic beats, median (IQR)	5.5 (1–25.5)

ACA, anti-centromere antibodies; ATA, anti-topoisomerase I antibodies; CRP, C-reactive protein; dcSSc, diffuse cutaneous systemic sclerosis; hsTnI, high-sensitivity troponin I; IQR, interquartile range; mRSS, modified Rodnan skin score; NT-proBNP, N-terminal pro-B-type natriuretic peptide.

### MVP score

At baseline, the MVP score had a median of 2.0 (IQR, 2.0–3.0), with values ranging from 1 to 4. In exploratory age- and sex-adjusted linear regression analyses, higher MVP scores were associated with the modified Rodnan skin score (mRSS; β = 0.422; *P* = 0.026), C-reactive protein (CRP; β = 0.412; *P* = 0.028) and NT-proBNP levels (β = 0.557; *P* = 0.003). No significant associations were observed with disease subset, patient-reported outcome measures (Sclero-ID, SHAQ), or the presence of atrial tachycardia. Detailed regression results are presented in Table 2.

**Table 2:** Age- and sex-adjusted linear regression analyses for factors associated with the MVP score (*n* = 34)

Predictor	R	Adjusted R^2^	B (95% CI)	β	*P*
mRSS	0.435	0.096	0.065 (0.008 to 0.121)	0.422	**0.026**
dcSSc	0.312	0.000	0.718 (−0.133 to 1.568)	0.315	0.095
CRP	0.401	0.071	0.107 (0.012 to 0.202)	0.412	**0.028**
NT-proBNP	0.519	0.191	0.003 (0.001 to 0.005)	0.557	**0.003**
Sclero-ID	0.165	−0.105	−0.006 (−0.051 to 0.039)	−0.060	0.780
SHAQ	0.136	−0.109	0.022 (−0.649 to 0.649)	0.014	0.945

Values in bold are statistically significant (*P* < 0.05). Linear regression adjusted for age and sex; separate models were fitted for each predictor. B, unstandardized regression coefficient; β, standardized regression coefficient; CI, confidence interval; CRP, C-reactive protein; dcSSc, diffuse cutaneous systemic sclerosis; mRSS, modified Rodnan skin score; MVP, morphology-voltage-P-wave duration; NT-proBNP, N-terminal pro-B-type natriuretic peptide; Sclero-ID, EULAR Systemic Sclerosis Impact of Disease; SHAQ, Scleroderma Health Assessment Questionnaire.

### Fragmented QRS

At baseline, fQRS was assessable in 32 patients; two limited cutaneous systemic sclerosis (lcSSc) recordings were excluded because the acquisition filter settings did not meet those specified in the study protocol. Of the 32 patients included in the fQRS analysis, 7 (21.9%) had QRS fragmentation. Of these, 6 had fragmentation in the inferior leads and 1 in the anterior leads. When stratified by disease subset, fQRS was present in 5/24 (20.8%) patients with lcSSc and 2/8 patients with diffuse cutaneous SSc (dcSSc) (25.0%), without a statistically significant difference between groups. No significant associations were observed between fQRS and clinical manifestations, biomarkers, patient-reported outcome measures, or antibody type. In exploratory age- and sex-adjusted logistic regression analysis, the number of PVCs was associated with the presence of fQRS (OR = 1.0022, 95%, CI 1.0001–1.0044; *P* = 0.044) (Table 3).

**Table 3:** Age- and sex-adjusted logistic regression for the presence of fragmented QRS at baseline

Predictor	OR (95% CI)	*P*
mRSS	0.932 (0.785–1.106)	0.421
Disease subset	0.705 (0.100–4.983)	0.726
Digital ulcers	1.207 (0.154–9.485)	0.858
Dysphagia	5.647 (0.832–38.316)	0.076
Sclero-ID score	1.058 (0.944–1.185)	0.331
SHAQ score	2.788 (0.579–13.425)	0.201
NT-proBNP	1.001 (0.995–1.006)	0.814
hsTnI	1.072 (0.714–1.608)	0.738
Anti-topoisomerase I antibodies	0.566 (0.091–3.499)	0.538
Anti-centromere antibodies	1.656 (0.237–11.576)	0.611
Number of PVCs	1.0022 (1.0001–1.0044)	**0.044**

Values in bold are statistically significant (*P* < 0.05). CI, confidence interval; hsTnI, high-sensitivity troponin I; mRSS, modified Rodnan skin score; NT-proBNP, N-terminal pro-B-type natriuretic peptide; OR, odds ratio; PVC, premature ventricular contraction; Sclero-ID, EULAR Systemic Sclerosis Impact of Disease; SHAQ, Scleroderma Health Assessment Questionnaire.

### Interatrial block

Partial IAB was observed in nine patients (26.5%) at baseline and advanced IAB in one patient. It was more frequent in patients with dcSSc (50.0%) than in those with lcSSc (19.2%), although the difference was not statistically significant. Patients with IAB had significantly higher mRSS values than those without IAB (9 ± 9 versus 3 ± 4, *P* = 0.040). Spearman analysis confirmed a positive correlation between mRSS and IAB (ρ = 0.381, *P* = 0.038).

In exploratory age- and sex-adjusted logistic regression analysis, mRSS was associated with IAB (OR = 1.187; 95% CI, 1.025–1.376; *P* = 0.022). NT-proBNP was also associated with IAB (OR = 1.009; 95% CI, 1.001–1.017; *P* = 0.027). No significant associations were observed for disease subset, antibody profile, physician global assessment, patient global assessment, or Sclero-ID score (Table 4).

**Table 4:** Age- and sex-adjusted logistic regression for the presence of partial interatrial block (*n* = 34)

Predictor	OR (95% CI)	*P*
mRSS	1.187 (1.025–1.376)	**0.022**
Disease subset	0.230 (0.039–1.358)	0.105
Digital ulcers	0.448 (0.066–3.023)	0.410
Dysphagia	1.336 (0.273–6.537)	0.721
Physician global assessment	1.057 (0.992–1.126)	0.089
Patient global assessment	1.003 (0.962–1.045)	0.887
Sclero-ID	0.910 (0.598–1.387)	0.662
NT-proBNP	1.009 (1.001–1.017)	**0.027**
Anti-topoisomerase I antibodies	1.768 (0.313–10.004)	0.519
Anti-centromere antibodies	1.510 (0.276–8.253)	0.635

Values in bold are statistically significant (*P* < 0.05). CI, confidence interval; mRSS, modified Rodnan skin score; NT-proBNP, N-terminal pro-B-type natriuretic peptide; OR, odds ratio; Sclero-ID, EULAR Systemic Sclerosis Impact of Disease.

## DISCUSSION

Systemic sclerosis is characterized by progressive inflammation, microvascular dysfunction, and fibrosis, processes that may affect the myocardium long before clinical symptoms become evident. Detecting early cardiac involvement remains challenging, particularly in asymptomatic patients. In this context, electrocardiography represents a simple and widely available tool that may provide information on electrical abnormalities.

In this study, we investigated the associations between the MVP score, fQRS, and IAB and clinical and biological features in patients with SSc.

The MVP score has emerged as a validated marker for predicting the occurrence of atrial fibrillation (AF) in different populations [18]. However, its application in SSc remains exploratory. A recent study analyzing ECG markers in elderly patients with elevated stroke risk found that patients with higher MVP scores, longer P-wave duration, and evidence of interatrial block on a standard 12-lead ECG had a greater likelihood of both atrial fibrillation and stroke [19]. Individuals with reduced P-wave voltage in lead I tended to develop AF more frequently, but this did not translate into an increased risk of stroke [8].

In our study, higher MVP values were associated with greater skin involvement, as reflected by the mRSS, as well as with elevated CRP and NT-proBNP levels. This suggests that ECG-derived atrial conduction abnormalities may coexist with systemic inflammation and markers of cardiac stress. MVP may therefore represent a marker associated with disease severity and cardiac stress, although its relationship with structural or functional cardiac involvement requires further investigation.

High-sensitivity troponin I is more closely associated with acute myocardial injury and may be released in inflammatory contexts, particularly during disease activation [20]. However, in our cohort, hsTnI values were within the normal range, suggesting that this biomarker did not capture evidence of myocardial injury.

Fragmented QRS was selected as an ECG marker of interest because it has been associated with myocardial fibrosis or scarring in various cardiac conditions [21,22]. Moreover, fQRS can be readily assessed on a standard 12-lead ECG. Given the central role of fibrosis in systemic sclerosis and the previously reported associations between fQRS and myocardial scarring in other cardiac conditions, fQRS may represent an ECG feature of potential interest in SSc [9,21,22]. However, myocardial fibrosis was not directly assessed in the present study, and no conclusions regarding the structural substrate of fQRS can be drawn from our findings.

Although not statistically significant, patients with a history of digital ulcers showed numerically higher odds of fQRS. This observation may suggest a potential link between peripheral microvascular disease and ECG abnormalities, an association suggested in previous studies reporting correlations between QTc prolongation and digital ulcers in SSc [23].

The number of ventricular extrasystoles on Holter ECG monitoring showed a significant association with the presence of fQRS after adjustment for age and sex, consistent with previous studies linking fQRS to ventricular arrhythmias [24,25].

Cardiac magnetic resonance imaging with late gadolinium enhancement represents the reference method for direct visualization of myocardial scar. However, its limited availability and higher cost restrict its use in routine clinical practice. Previous studies have shown that abnormal late gadolinium enhancement in various cardiac conditions is associated with the presence of fQRS on surface ECG [26,27].

Interatrial block has emerged as a marker of atrial arrhythmias, particularly atrial fibrillation and flutter, and has been associated with left atrial fibrosis [10,28]. In this study, the severity of skin involvement, quantified by mRSS, was associated with the presence of partial interatrial block. This finding suggests an association between greater skin severity and atrial conduction abnormalities. However, direct evidence of atrial fibrosis was not available, and the cross-sectional design does not allow conclusions regarding the temporal or pathophysiological relationship between these findings.

In addition, NT-proBNP levels were associated with the presence of IAB, supporting the hypothesis that subtle functional cardiac changes may coexist with electrical alterations detectable on surface ECG. Similar observations have been reported in other conditions characterized by myocardial fibrosis or infiltration, in which NT-proBNP levels were associated with atrial conduction abnormalities [29].

The present study has several limitations. First, the sample size was relatively small and the study was conducted at a single center. The extensive exclusion criteria resulted in a highly selected cohort of patients with SSc without overt cardiac disease or major comorbidities that could influence the ECG findings. This approach was intentionally adopted to reduce potential confounding and to explore the ECG-derived associations in a relatively homogeneous population; however, it limits the generalizability of our findings. In addition, the predominance of patients with lcSSc in our cohort may partly explain the relatively low median mRSS and the restricted range of skin involvement, and should be considered when interpreting associations involving mRSS.

Given the limited sample size and the low number of events for some outcomes, the regression analyses should be considered exploratory and hypothesis-generating. The small number of outcome events, particularly for fQRS and IAB, further limits the stability and precision of the logistic regression estimates. These results should therefore not be interpreted as confirmatory evidence of independent associations. The resulting estimates, particularly those with borderline statistical significance, may be sensitive to small variations and should be interpreted with caution. As no adjustment for multiple comparisons was performed in this exploratory study, the possibility of type I error should also be considered. Confirmation in larger cohorts is warranted.

Second, adjustment was limited to age and sex in order to preserve model stability in this exploratory cohort; residual confounding from other cardiovascular risk factors, disease-related characteristics and treatments therefore cannot be excluded. ECG interpretation was primarily performed by a single cardiologist, and intra- and inter-observer agreement was not formally assessed. Finally, cardiac MRI was not performed, limiting our ability to evaluate myocardial fibrosis directly and to further characterize the structural basis of the observed ECG abnormalities. These findings should therefore be interpreted as preliminary and require confirmation in larger prospective studies incorporating advanced cardiac imaging.

## CONCLUSION

In this exploratory study of a highly selected cohort of patients with SSc without overt cardiovascular disease, ECG-derived markers such as the MVP score, fragmented QRS and interatrial block were associated with selected clinical and biological characteristics. These findings should not be interpreted as evidence of structural cardiac involvement or myocardial fibrosis. Given the cross-sectional design, no temporal or predictive relationships can be established. Larger prospective studies integrating ECG markers with advanced cardiac imaging and clinical outcomes are needed to clarify their relationship with cardiac involvement in systemic sclerosis.

## Data Availability

Further data are available from the corresponding author on reasonable request.
